# A cohort study on the incidence and outcome of pulmonary embolism in trauma and orthopedic patients

**DOI:** 10.1186/1741-7015-12-39

**Published:** 2014-03-04

**Authors:** Suribabu Gudipati, Evangelos M Fragkakis, Vincenzo Ciriello, Simon J Harrison, Petros Z Stavrou, Nikolaos K Kanakaris, Robert M West, Peter V Giannoudis

**Affiliations:** 1 Academic Department of Trauma and Orthopaedics, School of Medicine, University of Leeds, Leeds General Infirmary, Clarendon Wing Level A, Great George Street, LS1 3EX Leeds, West Yorkshire, UK; 2 Leeds Institute of Health Sciences, University of Leeds, 101 Clarendon Road, LS2 9LJ Leeds, West Yorkshire, UK; 3 Leeds Biomedical Research Unit, Chapel Allerton Hospital, LS7 4SA Leeds, West Yorkshire, UK

**Keywords:** Pulmonary embolism, Deep venous thrombosis, Trauma, Orthopedic surgery, Arthroplasty, Mortality, Incidence

## Abstract

**Background:**

This study aims to determine the incidence of pulmonary embolism (PE) in trauma and orthopedic patients within a regional tertiary referral center and its association with the pattern of injury, type of treatment, co-morbidities, thromboprophylaxis and mortality.

**Methods:**

All patients admitted to our institution between January 2010 and December 2011, for acute trauma or elective orthopedic procedures, were eligible to participate in this study. Our cohort was formed by identifying all patients with clinical features of PE who underwent Computed Tomography-Pulmonary Angiogram (CT-PA) to confirm or exclude the clinical suspicion of PE, within six months after the injury or the surgical procedure.

Case notes and electronic databases were reviewed retrospectively to identify each patient’s venous thromboembolism (VTE) risk factors, type of treatment, thromboprophylaxis and mortality.

**Results:**

Out of 18,151 patients admitted during the study period only 85 (0.47%) patients developed PE (positive CT-PA) (24 underwent elective surgery and 61 sustained acute trauma). Of these, only 76% of the patients received thromboprophylaxis. Hypertension, obesity and cardiovascular disease were the most commonly identifiable risk factors. In 39% of the cases, PE was diagnosed during the in-hospital stay. The median time of PE diagnosis, from the date of injury or the surgical intervention was 23 days (range 1 to 312). The overall mortality rate was 0.07% (13/18,151), but for those who developed PE it was 15.29% (13/85). Concomitant deep venous thrombosis (DVT) was identified in 33.3% of patients. The presence of two or more co-morbidities was significantly associated with the incidence of mortality (unadjusted odds ratio (OR) = 3.52, 95% confidence interval (CI) (1.34, 18.99), *P* = 0.034). Although there was also a similar clinical effect size for polytrauma injury on mortality (unadjusted OR = 1.90 (0.38, 9.54), *P* = 0.218), evidence was not statistically significant for this factor.

**Conclusions:**

The incidence of VTE was comparable to previously reported rates, whereas the mortality rate was lower. Our local protocols that comply with the National Institute for Health and Clinical Excellence (NICE) guidelines in the UK appear to be effective in preventing VTE and reducing mortality in trauma and orthopedic patients.

## Background

Pulmonary embolism (PE) and deep venous thrombosis (DVT) can be considered under the spectrum of venous thromboembolic (VTE) disease. No definitive scientific data exist regarding the overall incidence of VTE in the general population, but a recent study estimates the incidence to range between 1 and 5/1,000 in the general population [1]. In the surgical population, the prevalence can reach more than 50% in the absence of thromboprophylaxis [1].

Worldwide, more than 50% of all hospitalized patients are at risk for VTE and surgical patients are at higher risk than medical patients [2]. The incidence of PE represents 5 to 10% of deaths in the hospital setting, making this condition the most common preventable cause of in-hospital death [3-6]. In addition, VTE and associated complications contribute substantially to patient morbidity and treatment costs [7,8].

Within the discipline of trauma and orthopedics, the prevalence of DVT and PE has been estimated to be 1.16% and 0.93%, respectively [9]. Mortality rates have been reported to range between 0.38% and 13.8% [10,11]. Principal risk factors include an injury severity score (ISS) greater than 50 and more than two surgical procedures [9]. PE appears to be the most common cause of mortality in patients that survive the first 24 hours following trauma and retrospective post-mortem data have demonstrated that out of an overall mortality of 13.8%, 1.6% was a consequence of fatal PE [10]. In the elective orthopedic clinical setting, PE is the second most frequent cause of death in patients that undergo lower limb total joint arthroplasty [11].

Despite the existing data reporting on the overall prevalence of PE in the trauma and orthopedic population, it remains a common belief that as the clinical signs and symptoms are non-specific and frequently silent, this complication may still be underdiagnosed [12]. The aim of this study is to determine the incidence of PE in trauma and orthopedic patients admitted to one of the largest tertiary referral centers in the UK and to investigate its association with the pattern of injury, type of treatment, co-morbidities, thromboprophylaxis and mortality.

## Methods

### Study design and setting, and study population

This cohort study was performed in a single center (a large UK teaching hospital - NHS trust). All patients admitted to our institution, from January 2010 to December 2011, for acute trauma or elective orthopedic procedures were eligible to participate in this study. Patients admitted for medical reasons or for other surgical causes not relevant to our discipline were excluded.

The study group of patients was formed by selecting all the patients who had clinical features suggestive of PE and who underwent subsequent radiological investigation (Computed Tomography Pulmonary Angiogram, CT-PA) to either confirm or exclude the clinical suspicion, within six months after the index orthopedic or acute trauma procedure. All patients gave written informed consent.

In our hospital, we use multidetector CT scanners (16- and 64-detector row). Higher specification machines are available, namely 128 and 320 slice, but the standard technique is similar. The main contraindications are renal failure and iodine allergy. In these cases a ventilation/perfusion (V/Q) scan is performed to confirm the diagnosis of pulmonary embolism. When an acute life threatening PE is suspected, a bedside echo looking for a right heart strain is also used. Further available options include pulmonary angiography and gadolinium enhanced MRI, but these are rarely performed or used due to their invasive nature and logistic difficulties.

CT-PA scans were considered as positive according to the following criteria: failure of contrast material to fill the entire lumen because of a central filling defect (the artery may be enlarged, as compared with similar arteries); a partial filling defect surrounded by contrast material on a cross-sectional image; contrast material between the central filling defect and the artery wall on an in-plane, longitudinal image; and a peripheral intraluminal filling defect that forms an acute angle with the artery wall [13,14]. A CT Venogram (CTV) was routinely performed in those patients that had a positive CT-PA.

PE is usually classified as proximal or distal depending on the location of the emboli identified on the CT scan. Usually when the emboli are located within the main or lobar arteries they have been reported as proximal PE and anything segmental or sub-segmental is usually reported as distal PE [15].

Institutional Board Review approval (Leeds Teaching Hospital NHS Trust) was obtained for this study (IBR number 10138).

### Data collection

Health records and electronic databases were further reviewed to identify a patient’s risk factors for developing VTE disease (according to the guidelines produced by NICE (National Institute for Health and Clinical Excellence httreatmentprotocol://guidance.Nice.org.uk/CG92; httreatmentprotocol://guidance. nice. Org.uk/CG46) [16].

Patient co-morbidities, the length of in-hospital stay, the characteristics of orthopedic interventions, the use of thromboprophylaxis (TP), the timing of PE diagnosis from the time of admission, as well as mortality, were all recorded. The severity of the PE episode was evaluated using the simplified Pulmonary Embolism Severity Index [17].

### Structure of thromboprophylaxis

All patients admitted to our institution, are expected to receive an initial risk assessment for VTE and have a specific form completed to identify risk factors, according to the current standard operating procedure of the Trust. This assessment tool has been developed in line with NICE guidelines [16]. This evaluation allows prescription of the most suitable mechanical and/or pharmacological TP treatment. This risk assessment is completed on admission and is reassessed during inpatient stay and adjusted according to the patient’s clinical condition. All patients are given a leaflet relevant to VTE and the measures that should be taken to minimize the risk of developing PE.

Patients undergoing elective total hip and knee arthroplasty surgery (THA, TKA) are treated with mechanical VTE prophylaxis (mechanical TP treatment), (anti-embolism stockings/alternative pneumatic devices from admission). Chemical thromboprophylaxis (chemical TP), with low-molecular weight heparin (LMWH) is provided post-operatively once the risk of bleeding is reduced (the wound is dry or the hemoglobin fall is <2 g/dl). Chemical TP treatment is continued for 35 days in patients undergoing THA, and 14 days for TKA [18-20]. With reference to other orthopedic procedures, including upper limb surgery, a TP treatment is not routinely prescribed, unless the patient is at risk of developing DVT/PE as highlighted in our risk assessment questionnaire tool. In these cases the patient is informed and application of mechanical VTE prophylaxis is considered. Administration of LMWH, 6 to 12 hours after surgery, is also considered. The administration of the mechanical VTE prophylaxis and LMWH is continued until the patient is fully mobile.

The TP treatment used for hip fractures is similarly based on patient risk assessment and starts with mechanical TP treatment. LMWH is administered once per day (usually tinzaparin 4,500 IU or enoxaparin 20 mg in patients with renal failure). Chemical TP treatment is interrupted 12 hours before surgery but restarts when the risk of bleeding is reduced and is continued for 28 days. Mechanical TP treatment is continued until the patient is fully ambulant [21].

Patients with major trauma or spinal injury routinely receive a mechanical TP treatment on admission. The risk of VTE and bleeding are also evaluated to determine the timing of initiation of a chemical TP treatment. The TP treatment is continued until mobility is fully restored (usually eight weeks for patients with pelvic and acetabular fractures). In cases where the risks of both VTE and bleeding are high and there is a previous positive PE history, a vena cava filter is inserted [22].

The use of lower limb plaster casts increases a patient’s risk for VTE. The patient is informed and a subsequent risk assessment is performed. LMWH treatment (tinzaparin 4,500 units or enoxaparin 20 mg in patients with renal failure) is prescribed for the whole time period of immobilization [23].

As a general measure, we recommend that all patients should, where possible, undergo early mobilization and regular exercises to minimize the risk of VTE.

### Statistical analysis

Continuous variables were summarized in terms of mean values with standard deviation and range as measures of variability. For variables that have skewed distributions, or otherwise may not be well represented by a normal distribution, medians and inter-quartile range are reported rather than mean and standard deviation. Categorical values were presented as absolute frequencies and percentages. Data were processed and analyzed by MedCalc version 12.2.1 (MedCalc software bvda, Mariakerke, Belgium).

Mortality following PE was modelled using a multivariable logistic regression on potential risk factors. These were assessed using a Wald test with a *P*-value of 0.05 or less considered to be statistically significant. Modelling was undertaken using the software development environment R version 3.0.0 [24]. It is noted that the analysis is exploratory only, to identify those factors most strongly associated with mortality. A variety of plausible models, including and excluding covariates, were explored and the final model presented is a parsimonious one, which includes only statistically significant predictors.

## Results

**Figure 1:**
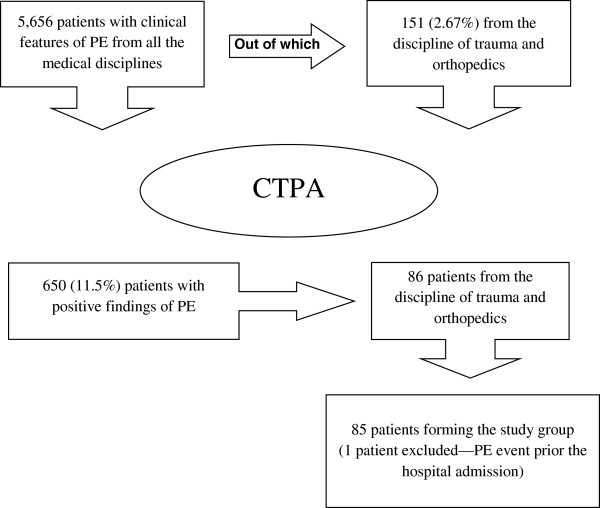
Data flow for patients included in the study group.

Over the pre-specified study period, 18,151 orthopedic patients (10,648 for elective operations and 7,503 for trauma) were admitted to our institution. During the same study period, 5,656 CT-PA investigations were requested by all the medical disciplines out of which 151 (2.67%) requests were from the discipline of trauma and orthopedics. Six hundred and fifty (11.5%) patients had positive findings of PE. Out of the positive 650 cases, 86 (13.2%) patients were from our discipline (trauma and orthopedics) and formed the study cohort. Taking into consideration the number of clinically suspected PE (151) and the number of positive findings on the CT-PA (86 patients) it was estimated that 57% of patients presenting with clinical features consistent for PE had positive radiological CT-PA findings. One patient, however, was excluded from the study as the PE event occurred shortly prior to hospital admission for TKA and, thus, 85 patients formed the study cohort (see Figure 1).

### Characteristics of the population

**Figure 2:**
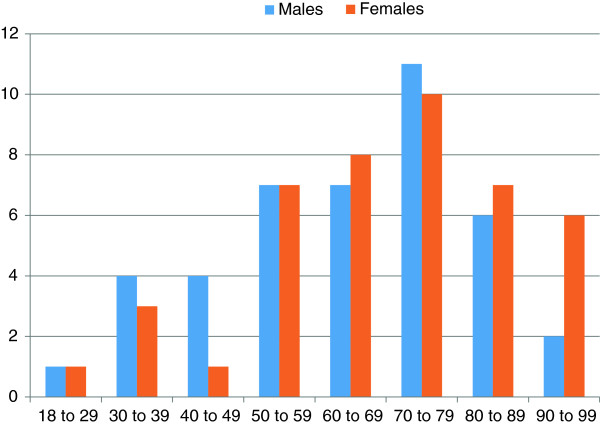
Age range and gender distribution vs number of patients (that is, frequency).

The mean age of patients was 66.3 years (range 18 to 98 years) and there was an almost equal gender distribution (M:F = 42:43). Dividing the age of patients into decades, the most populated categories were 50 to 59 years (16.5%), 60 to 69 years (17.6%), 70 to 79 years (24.7%), 80 to 89 years (15.3%) (Figure 2).

### Type of injuries and surgical procedures

Out of the 85 patients, 24 (28.2%) underwent elective orthopedic procedures with the most frequent being THA and TKA. Less common procedures included knee arthroscopy and spinal surgery. Sixty-one patients were admitted following trauma. Of these 61 patients with traumatic injury, 11.9% of them had sustained multiple injuries. The vast majority of the injured patients sustained lower limb trauma (Tables 1 and 2).

**Table 1:** Frequency, mortality and time of death

**Elective**					
**Type of procedure or injury**	**Number of patients**	**%**	**Mortality (N),%**	**Time to death after PE diagnosis**	
Total hip arthroplasty	6	7%	0		
Total knee arthroplasty	10	11.8%	0		
Spine	3	3.5%	(2) 2.4%	112	
Knee arthroscopy	3	3.5%	0		
Femoral osteotomy	1	1.2%	0		
Metalwork removal	1	1.2%	0		
Sum	24	28.2%	(2) 2.4%	Median: 112	
**Trauma**					
**Type of procedure or injury**	**Number of patients**	**%**	**Mortality (N),%**	**Time of death after PE**	
Femoral neck fixation	9	10.6%	(4) 4.7%	36	(5.9%) 21 days
Hemiarthroplasty	5	5.9%	(1) 1.2%	6	
Other lower limb injury	35	41.2%	(4) 4.7%	21	
Upper limb injury	9	10.5%	(2) 2.4%	134.5	
Pelvis and acetabulum	2	2.4%	0		
Vertebral fracture	1	1.2%	0		
Sum	61	71.8%	(11) 12.9%	Median: 36	
Entire PE cohort	85	100%	(13) 15.3%	Median: 69	

The time elapsed between the episode of PE and the death of patients, expressed in days, are also shown. PE, pulmonary embolism.

**Table 2:** Lower limb injuries details: type of injury and relative mortality

**Type of lower limb injuries**	**Pat. N**	**Death**
Femoral neck death	5	(5.9%)
Femoral diaphyseal fracture	2	0
Distal femur fracture	3	0
Tibial plateau fracture	9	(1) 1.2%
Tibial diaphyseal fracture	1	0
Ankle fracture	11	(1) 1.2%
Foot fracture death	2	(2.4%)
Multiple lower limb		
Fractures	5	0
Total meath	9	(10.7%)

Pat. N**,** number of patients.

### Co-morbidities and risk factors for VTE

Most of the patients had multiple comorbidities/risk factors. Because of the presence of more than one risk factor in each patient, the overall combination is more than 100%. More than four co-morbidities were present in 8 patients (9.4%), three in 20 patients (23.5%) and two in 26 patients (30.6%). The prevalence of known common risk factors for VTE and co-morbidities in the study cohort were: hypertension (36.8%), obesity (35.5%), cardiac disease (31.6%) and vascular disease (23.7%). The mean Charlson Co-morbidity Index was 1.7 (range: 0 to 10) and was greater or equal to 3 in 23 (30.26% of) patients (Table 3).

**Table 3:** Distribution of co-morbidities and VTE risk factors

**Risk factor**	**Pat. N.**	**%**
Malignancy	12	14.1%
Previous VTE	10	11.8%
Diabetes	10	11.8%
Chronic obstructive		
Pulmonary disease	13	15.3%
Cardiac disease	24	28.2%
Obesity	27	31.8%
Hypertension	28	32.9%
Vascular disease	18	21.2%
Thrombophylic disorders	1	1.2%
Contraceptives	3	3.5%
Hormonal therapy	4	4.7%
Varicose veins	2	2.4%
Rheumatic disease	5	5.9%
More than two comorbidities	26	30.6%
More than three comorbidities	20	23.5%
More than four comorbidities	8	9.4%
	Median	Range
Charlson Comorbidity Index	2 IQR = 0 to 3	0 to 10

Pat. N**,** number of patients; VTE, venous thromboembolism.

%**,** prevalence of the co-morbidity in the cohort.

Charlson Co-morbidity index is also shown.

### Thromboprophylaxis

Thromboprophylaxis was prescribed and administered in 65 cases (76.5%), representing the “prescribed TP” group. Aspirin alone was administered in six patients, which was not considered as an appropriate thromboprophylactic agent; thus, these cases were included to the “non-prescribed TP” group for all subsequent analysis. LMWH was used in the majority of cases (69.4%) (Table 4).

**Table 4:** Characteristic of thromboprophylaxis

**Treatment protocol yes**	**Pat. N.**	**%**
Number of patients	65	76.5%
**Type of TP treatment:**		
LMWH	59	69.4%
Mechanical TP	4	4.7%
ICF	2	2.4%
**Treatment protocol none**	**Pat. N.**	**%**
Number of patients	20	23.5%
**Causes:**		
Minor surgery	7	8.2%
Incomplete risk evaluation	11	12.9%
Refuse treatment	2	2.4%

ICF, Inferior vena cava filter; LMWH, low molecular weight heparin; Pat. N., patient number; TP, thromboprophylaxis.

Within the 65 patients receiving TP, 4 (4.7%) received mechanical TP only (two of these patients underwent TKA and developed above knee DVT). In two cases (2.4%) an inferior vena cava filter was inserted for recurrent episodes of VTE and in one case the filter was left *in situ* permanently.

Out of the 20 cases that did not receive TP, 7 (8.2%) patients did not have TP prescribed on account of undergoing a minor orthopedic procedure (three patients underwent knee arthroscopy, four patients sustained upper limb injuries (radial head fracture, rotator cuff tear, wrist and clavicle fracture). Out of the remaining 13 (15.3%) trauma patients, 11 patients did not receive TP (incomplete evaluation of patient risk profile and partial ambulation of patient), whereas 2 patients refused treatment. Two patients from the trauma group were treated operatively (intra-capsular neck of femur fracture, femoral osteotomy with an Ilizarov frame), whereas the remaining 11 were managed non-operatively with plaster of Paris and brace application (1 Achilles tendon rupture, 4 ankle fractures, 1 ankle sprain, 4 metatarsal fractures, 1 tibial plateau fracture).

### Thromboembolic events

The overall incidence of PE was 0.46% (0.8% in the trauma cohort and 0.18% in elective orthopedic interventions). The median time of PE diagnosis, from the date of injury or the surgical intervention was 23 days (range 1 to 312). Only one patient had a very late diagnosis of PE (after 312 days), because he had a DVT three months after trauma to the left foot (fracture of the base of the fifth metatarsal treated conservatively by cast immobilization) and subsequently developed PE.

Out of the 85 patients forming the study cohort, there were no recorded cases of hemodynamic instability. All patients had a recorded systolic blood pressure of more than 100 mmHg at the time of onset of the clinical symptoms suspicious for PE. Only two patients were found collapsed with a respiratory problem, but the recorded vital parameters were all stable except for respiratory rate. One patient presented with an atypical presentation of gradual worsening of shoulder tip pain for a week following a muscle biopsy for a myositis and was confirmed to have PE on CT-PA.

The most common signs and symptoms observed were pleuritic chest pain, dyspnea, acute tachycardia and hypoxia. Elevation of the D-dimer value was also commonly observed. The mean simplified Pulmonary Embolism Severity Index was 2.27 (SD = 1.14). The extension and localization of the PE are shown in Table 5.

**Table 5:** Thromboembolic events description

**Location of PE**	**Extension**	**Pat. N.**
Right lung	Proximal	9 (10.6%)
	Distal	20 (23.5%)
	Proximal to distal	2 (2.4%)
Left lung	Distal	7 (8.2%)
Bilateral	Distal	14 (16.5%)
	Proximal	21 (24.7%)
	Proximal to distal	3 (3.5%)
	Multifocal	4 (4.7%)
	Proximal left and distal right	4 (4.7%)
	Proximal right and distal left	1 (1.2%)
**Total**		85
Concomitant DVT: extension		Pat. N
Proximal to the knee		11 (12.9%)
Distal to the knee		6 (7%)
Above and below the knee		11 (12.9%)
**Total**		28 (32.9%)

DVT, deep vein thrombosis; Pat. N., patient number; PE, pulmonary embolism.

Concomitant DVT was present in 28 patients (32.9%) with PE. Proximal DVT was observed in 11 patients, and in 6 patients distal DVT was observed. In the remaining 11 patients a DVT was identified both proximal and distal to the knee joint (Table 5).

### Hospitalization details

Within the study cohort the mean time for hospital stay was 18.5 days (range 1 to 64 days). In 33 cases (39%), PE developed during the in-hospital stay. In the remaining cases, PE developed following hospital discharge necessitating re-admission of these patients for treatment. The mean length of stay (LOS) for PE re-admission was 8.5 days (range 1 to 28 days). In comparison to the same uncomplicated orthopedic surgical procedures, the onset of PE led to an increase in the LOS, with a mean rise of 2.4-fold in hospitalization time.

### Mortality

The overall inpatient mortality for the whole hospital cohort was 2.3% (419 out of 18,151 patients). Mortality after PE was 0.07% (13 out of 18,151 patients) reaching 15.3% (13 out of 85) of the patients with a positive CT-PA (Tables 1 and 2).

Out of the 13 patients who died, 7 underwent surgery (5 proximal femur fracture and 2 for a vertebral metastatic lesion) and 6 non-operative treatment (1 tibial plateau, 2 metatarsal, 1 clavicle, 1 radial head and 1 ankle fracture) (Tables 1 and 2).

For elective orthopedic procedures, the rate of mortality was 0.02%, whereas in the trauma population it was 0.15%. Two patients (tibia plateau and metatarsal injury) out of the 13 who died did not receive a TP treatment although subsequent risk assessment classified them as high-risk patients. These two patients were managed non-operatively with a cast immobilization. Risk factors identified included previous history of DVT, malignancy, congestive pulmonary disease and obesity.

Of the 13 patients who died, only 1 patient’s primary cause of death was certified to be PE. The primary causes of death, as defined in the death certificate obtained from the coroner for those who died in the hospital and from the office of national statistics for those who died at home, and certified by the doctor after discussion with the coroner, are presented in Table 6.

**Table 6:** Causes of death

**No**	**Gender**	**Age**	**Cause of death**
1	M	80	Lung cancer
2	M	64	Disseminated lung cancer
3	F	96	Bronchopneumonia
4	F	88	CCF, IHD*
5	M	90	CCF, IHD*
6	F	88	Spontaneous subdural hematoma
7	F	90	PE*, Bilateral DVT
8	F	63	Bronchopneumonia
9	M	60	Metastatic lung cancer
10	F	84	Aspiration pneumonia, stroke
11	F	79	Bronchopneumonia
12	M	64	Metastatic renal cancer
13	F	80	Pulmonary fibrosis

*; CCF, congestive cardiac failure; DVT, deep venous thrombosis; IHD, ischemic heart disease; PE, pulmonary embolism.

In the trauma cohort the median time of death from PE diagnosis was 36 days (range 5 to 151), whereas for the elective orthopedic group it was 112 days (84 to 140).

The presence of two or more co-morbidities was significantly associated with the incidence of mortality (unadjusted OR = 3.52, 95% CI (1.34, 18.99), *P* = 0.034). Although there was also a similar clinical effect size for polytrauma injury on mortality (unadjusted OR = 1.90 (0.38, 9.54), *P* = 0.218), evidence was not statistically significant for this factor. We found little evidence of association with gender, time of surgery, elective/trauma or the proximal/distal position of embolism. For the following factors, however, there was strong evidence (statistically significant at the 5% level) expressed below as odds ratios with 95% confidence intervals. Most importantly, there was a strong associated risk of mortality following pulmonary embolism in patients with high Charlson’s comorbidity index and the usage of thromboprophylaxis was seen to be associated with a protective effect. Results are given in Tables 7 and 8.

**Table 7:** Regression coefficients expressed as ORs

**Covariate or factor**	**Unadjusted OR (95% CI)**	**Adjusted OR (95% CI)**	***P*-value(adjusted)**
Age per year	1.05 (1.01, 1.10)	1.06 (1.00, 1.12)	0.05
Charlson per comorbidity	1.58 (1.20, 2.09)	2.02 (1.30, 3.16)	0.01
Prophylaxis (other than aspirin)	0.29 (0.08, 1.05)	0.06 (0.01, 0.48)	0.01

**Table 8:** Characteristics of patients after six months of development of PE

	**Alive at six months**	**Died within six months**	**All**
Number	74	11	85
Age, mean ± SD (yrs)	64.6 ± 17.9	77.7 ± 13.2	66.3 ± 17.8
Gender M/F	38/36	5/6	43/42
Charlson, median; IQR	1:2	3:5.5	1:3
Thromboprophylaxis Y/N	60/19	5/6	65/20
Time surgery or admission to PE (days), median; IQR	18:30	23:29	19:30
Elective/Trauma	22/52	2/9	24/61
Proximal/Distal	33/36	4/4	37/40

PE, pulmonary embolism.

One more interesting observation we found in our study was the association of mortality with age. From the 85 patients that developed PE following orthopedic surgery, 11 died within six months. There was a statistically significant trend recorded that found older patients more likely to die following a PE after orthopedic/trauma admission.

Also, the presence of DVT was associated with increased mortality following a PE after an orthopedic/trauma admission. This association between DVT and mortality was not statistically significant in this dataset.

## Discussion

Despite continuous improvement in medical knowledge and treatment modalities, the incidence of VTE and its related complications has remained fairly static during the last three decades [25]. Notwithstanding the implementation of prevention protocols, clinical manifestation of PE is not clear or specific. PE may be expressed in a silent way and can be missed by the clinical team. For this reason, PE might be under diagnosed.

In our study population, the incidence of PE was in line with data published in previous studies [1,9,12,26-28]. On further subgroup analysis it was noted that the incidence of PE was lower for the elective surgery cohort (0.23%) compared to the data recently published in the guidelines of the American College of Chest Physicians [26] (0.35%) and Markovic-Denic *et al*. [1] (1.6%), but is comparable to that reported by Jean-Marie Janue (0.14% in THA and 0.27% in TKA) [12]. The prevalence noted in trauma patients was in accordance with Maneker *et al*. [29] who showed a rate of 0.27% in his study population, which is lower than the rates reported by other studies [9,30]. These differences, nevertheless, are difficult to explain in retrospective studies, performed in different geographic areas with different protocols of prophylaxis and treatment.

On the basis of current evidence, CT-PA is considered the gold standard for the diagnosis of PE [13,31]. Chest contrast enhanced CT replaced catheter angiography due to its less invasive nature and accuracy, and has been proven to be superior or equal to angiography [31]. The reported sensitivity for the diagnosis of PE with CT-PA varies from 45 to 100% and the specificity from 78 to 100% [31]. CT-PA has some limitations in detecting isolated sub-segmental PE [31], but the introduction of the multi-detector CT technique currently allows evaluation of pulmonary vessels down to the sixth order branches, thus, significantly increasing the rate of detection of PE (sensitivity: 83%, specificity: 96%) [14,32].

Many authors have attempted to correlate specific risk factors to the development of PE. Strong correlation was identified for the number and magnitude of surgical interventions, previous history of VTE and the length of the hospitalization period [25,33,34]. The next highly reported risk factors for VTE are cardiovascular disease [1,23,33,34] and obesity [10,12,23,35]. More than half of our study cohort belongs to the high-risk category with more than two risk factors being present as defined by the NICE guidelines [16].

Although 79.3% of our study population was on TP treatment, patients still developed PE. We could not identify any additional specific related factors to VTE development, but we have observed that in our cohort, 62.4% of patients were older than 60 years and 22.4% were more than 80 years of age. Several studies reported age as an independent risk factor for VTE [1,26,36]. In our cohort, many of these elderly patients also had lower limb pathology (83.5%). One may speculate a synergistic effect of these two parameters in reducing mobility and leading to a higher risk of developing PE. In a recent case-crossover study reduced mobility was reported as a significant trigger of hospitalization for VTE. The risk of VTE hospitalization was 4.2-fold greater in the time period when reduced mobility occurred [37].

In 13 cases, TP treatment was not prescribed even though the patients had risk factors for VTE. Eight of these 13 cases sustained foot and ankle injuries, which were managed non-operatively and followed up in the outpatient fracture clinics. The reason for this can be attributed to the lack of clarity of national and local guidelines about TP treatment in the out-patient setting, particularly with injury patterns that are considered as less debilitating. Shibuya *et al*. [38] stated that routine use of TP treatment in foot and ankle injuries is not warranted in contrast to our findings, which support the view that even minor foot injuries cannot be neglected and risk assessment should be performed on an individual basis. This has led to the expansion of the routine risk assessment of patients treated in our out-patient setting and regular audit cycles have been implemented to ensure consistent compliance.

Concomitant DVT was identified in one-third of our study cohort. Knudson *et al*. [39] analyzed the American College of Surgeons National Trauma Data Bank and found 522 cases of PE out of 450,375 trauma patients (0.11%). In only 16% of these cases a concomitant DVT was diagnosed. In a prospective cohort study [40] of 397 patients with the clinical suspicion of PE, 149 were positive for PE and less than one-third had a concomitant DVT. Cipolle *et al*. [41] performed a trauma registry analysis of 10,141 trauma admissions, and found 30 cases of PE, of which only 5 (16.7%) had coexisting DVT. Moreover, in a retrospective review of medical records of 247 trauma patients who underwent TPA/CTV over a three-year period, Velmahos *et al*. [42] recognized positive findings of PE in 46 patients (19%) and among these, only 7 (15%) also had a DVT. Hypothesizing that CTPA/CTV was considered the most accurate method for diagnosing VTE, the same authors [42] investigated this lack of association between PE and DVT. They stated that it was unlikely that a diagnosis of DVT could have been significantly missed for an insufficient sensitivity of the diagnostic tool. Therefore, they hypothesized that the clots might be formed *de novo* within the pulmonary circulation as a consequence of the changes within the lung vascular endothelium and in the rheological blood properties induced by a post-traumatic hyper-adrenergic and hyper-inflammatory state. However, there is still no definitive evidence about the etiological relationship between DVT and PE, and further studies are desirable to comprehend this phenomenon.

Our mortality was low and consistent with other reports in the literature (Table 9). The low mortality rate noted in the trauma patient subgroup could be attributed to the high percentage of less severe traumatic injuries (frequency of polytrauma patients: 11.9%) and to the good compliance rate of implementing our TP treatment protocols. Consequently, the average number of co-morbidities in our sample was 2.6. A higher proportion of non-survivors had three or more co-morbidities in contrast to the survivors, and this difference was statically significant (*P* = 0.034).

**Table 9:** Literature review on the incidence and mortality of PE

**Elective**				
**Author**	**Year**	**Type**	**Incidence of PE**	**Mortality**
Januel JM *et al*. [12]	2012	Meta-analysis	THA: 0.14%	
			(0.07% to 0.21%)	
			TKA: 0.27%	
			(0.16% to 0.38%)	
Falck-Ytter Y *et al*. [26]	2012	Guidelines	THA: 0.3%	
Markovic-Denic L *et al*. [1]	2012	Prospective	THA: 1.6%	1.5%
			TKA: 1.5%	
Pedersen AB *et al*. [34]	2011	Retrospective	TKA: 0.3%	
Pedersen AB *et al*. [33]	2010	Retrospective	THA: 0.3%	1% (0.05% PE rel)
*Poultsides LA *et al*. [11]	2012	Meta-analysis		0.38% (0.2 to 0.59)
Our study			0.2%	0.02%
**Trauma**				
**Author**	**Year**	**Type**	**Incidence of PE**	**Mortality**
Jawa RS *et al*. [27]	2011	Retrospective	0.5%	
Paffrath T *et al*. [9]	2010	Retrospective	0.9%	
McNamara I *et al*. [30]	2009	Prospective	0.7%	
Huseynova K *et al*. [28]	2009	Retrospective	0.7%	
*Ho KM *et al*. [10]	2010	Retrospective		13.8% (1.6% PE rel)
Our study			0.8%	0.15%

*Study reporting PE-related mortality. PE, pulmonary embolism; THA, total hip arthroplasty; TKA, total knee arthroplasty.

The data have been compared with those of the current study.

The present study has several limitations, including the retrospective nature of data collection, from the case notes and electronic databases, the small sample size, the short study period (two years) and the absence of a control group. Moreover, our data pool documented events occurring only during hospital stay (primary or readmission). We are aware that some of our study population could have been admitted or treated elsewhere as we treat a number of tertiary referred patients and, as such, we might have missed some patients who developed PE. Strengths of the study include the identification of consecutive patients with specific injury patterns and risk factors who sustained PE in a large teaching hospital over a two-year period.

## Conclusions

In this study, following the NICE guidelines for thromboprophylaxis, the incidence of VTE was found to be similar to the rates reported in the international literature, whereas the mortality rate was considerably lower. It appears that local protocols, in compliance with the NICE guidelines, are effective in the prevention of VTE and in reducing mortality in trauma and orthopedic patients. However, despite the wide administration of both mechanical and chemical TP treatment, patients can still develop PE. It appears that the possibility of PE development is not only related to certain patient related risk factors but also to the consequence of all the aspects of the post-traumatic or post-surgical disease process. Overall, the type of treatment, the type and length of drug administration, the duration of immobilization and the individual response of each patient appear to contribute to the development of this rare yet fearful complication.

Further studies are desirable to monitor the incidence and outcome of PE in trauma and orthopedic patients so that on-going vigilance and on-going evaluation of the efficacy and effectiveness of treatment protocols will ensure that the morbidity will remain low and the mortality will continue to improve.

## Abbreviations

CT-PA: Computed Tomography-Pulmonary Angiogram; CTV: Computed Tomography Venogram; DVE: Deep venous embolism; DVT: Deep venous thrombosis; ISS: Injury Severity Score; LMWH: Low Molecular Weight Heparin; LOS: Length of stay; MTP: Mechanical thromboprophylaxis; NICE: National Institute for health and Clinical Excellence; PE: Pulmonary embolism; THA: Total Hip Arthroplasty; TKA: Total Knee Arthroplasty; TP: Thromboprophylaxis; VTE: Venous thromboembolism.

## Competing interests

No benefits have been received in any form by any of the authors with regard to the preparation of the manuscript. All authors declare that there is no conflict of interests.

## Authors’ contributions

SG participated in data collection, analysis, initial draft of the manuscript, revisions and prepared the final manuscript. EMF participated in data collection, analysis and initial draft of the manuscript. VC participated in data collection, statistical analysis and initial draft of the manuscript. SJH contributed to data collection and initial editing of the manuscript. PZS did the statistical analysis. NKK contributed to the study concept and design, and critical review of the final draft. RMW participated in the statistical analysis and final preparation of the manuscript. PVG contributed to the study concept and design, coordination of all the aspects of the study, critical revision of the manuscript, and administrative, technical and material support. All authors read and approved the final manuscript.

## Pre-publication history

The pre-publication history for this paper can be accessed here:

http://www.biomedcentral.com/1741-7015/12/39/prepub
